# Whoever wants better healthcare simply pays more: citizens' perception about voluntary private health insurance in Colombia

**DOI:** 10.1186/s12939-023-02086-z

**Published:** 2024-01-12

**Authors:** Daniel Felipe Patiño-Lugo, Claudia Marcela Vélez, Diana Patricia Díaz-Hernández, Olga Francisca Salazar-Blanco, Juan Esteban González-Arango, Juan Carlos Velásquez-Correa, Leydi Camila Rodríguez-Corredor, Viviana María Vélez-Marín, Pamela Velásquez-Salazar

**Affiliations:** 1 Unit of Evidence and Deliberation for Decision Making – UNED, Faculty of Medicine, University of Antioquia, Carrera 51D #62-42, Medellín, Antioquia Colombia; 2 Faculty of Medicine, University of Antioquia, Cra. 51D #62-29, Medellín, Antioquia Colombia; 3 Health Rehabilitation Research Group, Faculty of Medicine, University of Antioquia, Medellín, Colombia; 4 EDUSALUD Research Group, Faculty of Medicine, University of Antioquia, Medellín, Antioquia Colombia; 5 Epidemiology Group, National Faculty of Public Health, University of Antioquia, Medellín, Antioquia Colombia

**Keywords:** For-profit insurance plans, Employer-sponsored health insurance, Social security, Private practice, Accessibility to health services, Equity in access to health services

## Abstract

**Objectives:**

To explore the perceptions that Colombians have about voluntary private health insurance plans (VPHI) in the health system to identify the tensions that exist between the public and private systems.

**Methods:**

A qualitative case study approach with 46 semi structured interviews of patients, healthcare workers, healthcare administrators, decision-makers, and citizens. Interviews were recorded, transcribed, anonymized, digitally stored, and analyzed following grounded theory guidelines.

**Results:**

We developed a paradigmatic matrix that explores how, in a context mediated by both the commodification of health and social stratification, perceptions about the failures in the public health system related to lack of timely care, extensive administrative procedures, and the search for privileged care led to positioning VPHI as a solution to these failures. The interviewees identified three consequences of using VPHI: first, the worsening of problems of timely access to care in the public system; second, higher costs for citizens translated into double payment for technologies and services to which they are entitled; third, the widening of inequity gaps in access to health services between people with similar needs but different payment capacities.

**Conclusions:**

These findings can help decision makers to understand citizens´ perceptions about the implications that VPHI may have in worsening equity gaps in the Colombian health system. It also shows, how VPHI is perceived as a double payment for services covered within social security plans and suggests that the perceived lack of timely access to care in the public systems and the fear that citizens have for themselves or their family members when using suboptimal healthcare are important drivers to purchase these private insurances.

**Supplementary Information:**

The online version contains supplementary material available at 10.1186/s12939-023-02086-z.

## Resumen

**Objetivos:**

Explorar las percepciones que tienen los colombianos sobre los planes de seguro de salud privados voluntarios (VPHI) en el sistema de salud para identificar las tensiones que existen entre los sistemas público y privado.

**Métodos:**

Un estudio cualitativo de caso con 46 entrevistas semiestructuradas a pacientes, trabajadores de la salud, administradores de salud, tomadores de decisiones y ciudadanos. Las entrevistas se grabaron, transcribieron y almacenaron de manera anónima. El análisis se hizo siguiendo conceptos de la teoría fundamentada.

**Resultados:**

Desarrollamos una matriz paradigmática que explora cómo, en un contexto mediado tanto por la mercantilización de la salud como por la estratificación social, las percepciones sobre las fallas en el sistema de salud público relacionadas con la falta de atención oportuna, procedimientos administrativos extensos y la búsqueda de atención privilegiada llevaron a posicionar los VPHI como una solución a estas fallas. Los entrevistados identificaron tres consecuencias del uso de los VPHI: primero, el empeoramiento de los problemas de acceso oportuno a la atención en el sistema público; segundo, mayores costos para los ciudadanos, traducidos en un pago doble por tecnologías y servicios a los que tienen derecho; tercero, el aumento de las brechas de equidad en el acceso a los servicios de salud entre personas con necesidades similares pero diferentes capacidades de pago.

**Conclusiones:**

Estos hallazgos pueden ayudar a los tomadores de decisiones a comprender las percepciones de los ciudadanos sobre las implicaciones que el VPHI puede tener en el empeoramiento de las brechas de equidad en el sistema de salud colombiano. También muestra cómo el VPHI se percibe como un pago doble por servicios cubiertos dentro de los planes de seguridad social y sugiere que la falta percibida de acceso oportuno a la atención en los sistemas públicos y el miedo que los ciudadanos tienen por sí mismos o por sus familiares cuando utilizan una atención sanitaria subóptima son factores importantes para adquirir estos seguros privados.

## Introduction

Law 100 from 1993 transformed the Colombian health system through market-oriented reforms, creating the General System of Social Security in Health [1], which introduced the participation of intermediate insurance companies (EPS, for its acronym in Spanish) and public and private for-profit health care institutions (IPS, for its acronym in Spanish). This system, primarily financed with mandatory contributions from employers and employees [2], allows citizens to purchase Voluntary Private Health Insurance (VPHI), also known as prepaid medicine in Colombia, while continuing to contribute to the public system. This institutional arrangement has not changed after several health system reforms, and it might not change soon given that the major health care reform that is being discussed at Congress at this moment states that the insurance companies that sell VPHI can continue to do so [3].

In the global context, VPHI can be classified into three types: complementary, which covers costs such as copayments; supplementary, which covers additional benefits that are not included by the public system; and duplicate, aiming to provide faster access to specialists and services that are available in the public system [4]. In Colombia, there is a duplicate VPHI to have faster access, a greater pool of providers and better comfort in healthcare institutions.

In Colombia, there are healthcare providers that exclusively offer services to those who have VPHI. The percentage of citizens who have VPHI increased from 4.8% to 8.2% between 2008 and 2017. Data from 2021 show that private insurance accounted for 8% of the total health expenditure [5]. This situation is not unique to Colombia; most countries in the Organization for the Cooperation and Economics Development (OCDE) have VPHI [4]. What is a unique situation is that the private companies that manage public health insurance are the same companies that offer and manage different duplicate voluntary private health insurance.

There are arguments both in favor of and against VPHI in health systems. Some of the contended benefits include decreased out-of-pocket expenses [6], reduced wait times within the public health system by transferring patients to the private system [7], and increased savings in the public system. The contended savings occur due to the reduced demand for publicly financed services while continuing mandatory contributions to the public system. Based on this argument, some stakeholders suggest increasing the number of people with VPHI to generate greater savings for the public system [8].

In contrast, some of the argued disadvantages of the VPHI include differential access to healthcare services based on private payment capacity, which can be considered unfair on the basis that access to healthcare should be based exclusively on individual health needs [9–11]. Additionally, VPHI creates a barrier to the universal provision of healthcare services when some professionals and healthcare institutions only serve those who have this insurance. This compromises the quality of healthcare and increases wait times within the public health system given that some specialists and healthcare workers decide to dedicate more time to the private system, further neglecting the needs and provision of services in the public sector [12, 13]. Some research has shown that health systems with high VPHI participation are negatively associated with the principles of universality, equity, accessibility, and quality of care [14–17].

These arguments about VPHI have important implications for citizens. Therefore, it is important that public policy decisions that affect the relationship between public and private healthcare systems take into consideration the values and preferences of citizens regarding the use of VPHI. In this research, we set out to explore the perceptions that Colombians have about VPHI to identify areas of tension that exist between the public and private systems and to generate information that contributes to public policy decisions on this topic.

## Methods

This study is part of a broader study that proposed to explore the perceptions that Colombians have about which technologies and services should be paid for with public resources from the health system. We used a qualitative embedded case study approach [18]. The case is limited to the perceptions of Colombians about VPHI, and the embedded units were groups of participants with different knowledge, interests, and values regarding the Colombian healthcare system.

### Participant selection

We intentionally selected fives groups of participants with different experiences and perspectives regarding the health system: 1) patients with chronic diseases or who have recently used healthcare services; 2) healthcare professionals; 3) healthcare institution administrators within the past 5 years; 4) decision-makers of institutions at the national, departmental, or municipal level; and 5) citizens who have not had recent contact with healthcare services, have not been patients, or caregivers to patients in the last 12 months, are not healthcare professionals, administrators or decision makers, or have familial relations with one (up to the second level of consanguinity).

We identified possible participants in each group by searching for heterogeneity by age, geographic representation, and gender-equal representation. A list of possible candidates was proposed by the research team and then were contacted and invited to the study by telephone, email, or in person using a preestablished message. In addition, we asked interviewees to identify other possible participants.

### Collection and analysis of information

We used semi-structured interviews for in-person and virtual encounters. The interview included general questions about VPHI as well as a question based on an example regarding private insurance and its consequences on timely care (see Appendix). After signing the consent form, the interviews were recorded, transcribed, anonymized, and then digitally stored.

Grounded theory guidelines were followed for the analysis of the data [19]. Transcribed data were analyzed using an open, axial, and selective coding approach, involving constant comparison, aiming to identify, develop, and connect the concepts that would form the building blocks of the explanations. First, through open coding, pairs of two researchers created provisional codes (i.e., descriptive words or short phrases) within each transcript that captured small units of data (i.e., words, phrases, or paragraphs) that described a thought, opinion, feeling, or experience reported by interviewees. In full-team research meetings codes were continuously compared across transcripts to explore and discuss commonalities and singularities among groups of participants [20]. Open coding analysis continued until no new information was added from individual transcripts.

**Fig. 1:**
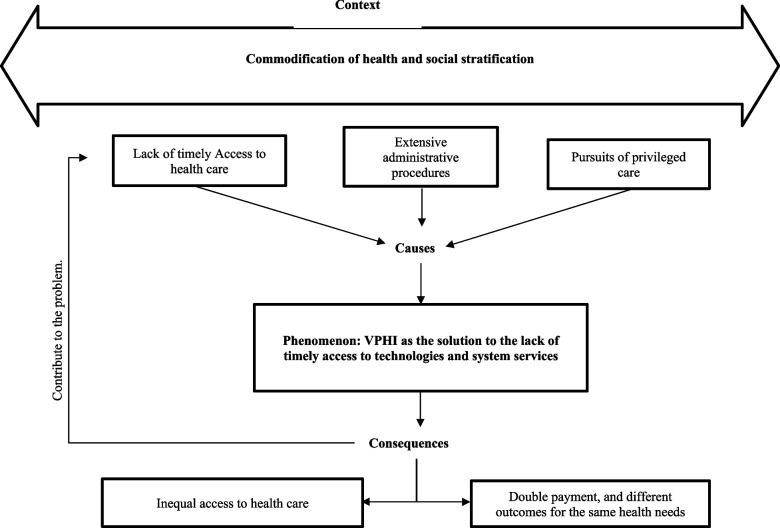
Paradigmatic matrix of citizens’ perceptions about VPHI in Colombia

The next step was axial coding, in this step, relations between codes were noted, intersecting codes were grouped together, and preliminary themes and subthemes depicting codes were identified to get meaning and identity to repeated opinions. Themes were created when they were shown to represent substantial concepts that connected extensive portions of the data together. In some cases, we developed subthemes that organized several concepts associated with a single theme. Subsequently, through selective coding, the central phenomenon (core category) was defined, along with themes and subthemes (i.e., context, causes, and consequences). Last, we diagrammed our findings into a paradigmatic matrix of citizens’ perceptions of VPHI in Colombia [20] (See Fig. 1). We stopped the interview process when no additional insights emerged during our analysis in the group meetings. An audit trail was documented as a strategy to establish the reliability of the data [21, 22].

### Reflexivity

In the research team meetings, there were discussions regarding personal experiences, opinions and knowledge about the health system. It was collectively confirmed that the emerging categories and connections between topics effectively emerged from the analysis of the interviews, and all identified viewpoints were represented both in the categories and in the findings to be reported.

## Results

### Description of the participants

We interviewed 46 people between the ages of 23 and 66 years old, with the majority being female (57%). The interviews were on average 43 min long, 39 were conducted virtually and seven were conducted in person. We interviewed five participants from each of the following groups: patients, healthcare services administrators and decision-makers; thirteen from the group of healthcare professionals; and eighteen from the group of citizens. Nine participants reported having VPHI: one patient, three citizens, two administrators, one decision maker, and two healthcare professionals. Participants resided in four departments of Colombia (i.e., Antioquia, Caldas, Meta, and Santander) and the district of Bogota. The general characteristics of the interviewees are summarized in Table 1 and Appendix.

**Table 1:** Participant characteristics by group

Sociodemographic characteristics		Citizens (18)	Patients (5)	Health professionals (13)	Administrators (5)	Decision makers (5)
**Variables**	**Categories**	**n (%)**	**n (%)**	**n (%)**	**n (%)**	**n (%)**
**Gender**	Female	11 (61,1)	4 (80,0)	7 (53,8)	2 (40,0)	2 (40,0)
	Male	7 (38,9)	1 (20,0)	6 (46,2)	3 (60,0)	3 (60,0)
**Health insurance scheme**	Subsidized (noncontributory) scheme for informal workers and low-income population	3 (16,7)	2 (40,0)	-(-)	-(-)	-(-)
	Contributory scheme (for employees and self-employed workers)	11 (61,1)	2 (40,0)	11 (84,6)	3 (60,0)	4 (80,0)
	Especial (for military and public employees)	1 (5,6)	-(-)	-(-)	-(-)	-(-)
	VPHI^a^	3 (16,7)	1 (20,0)	2 (15,4)	2 (40,0)	1 (20,0)
**Department**		Antioquia 14 (77,7) Bogota 3 (16,6) Caldas 1 (5,5)	Antioquia 3 (60,0) Santander 1 (20,0) Meta 1 (20,0)	Antioquia 7 (53,9) Bogota 2 (15,4) Santander 4 (30,7)	Antioquia 5 (100)	Antioquia 5 (100)
**Age range (in years)**		29–66	25–59	23–57	31–46	37–55

^a^*VPHI* Voluntary private health insurance All people with VPHI are mandatory affiliated to any of the other public schemes

In the group of patients, we interviewed people with a variety of conditions that required attention in clinical medicine, surgical, hospitalization and outpatient care services, and it included one participant who was the caregiver of a child who required recent medical care. In the group of health professionals, we included professionals from medicine, nursing, dentistry, social work, and surgical instrumentation, seven of them were working in public institutions, and six in private institutions. Health services administrators worked for different healthcare institutions or for municipal health secretariats. All decision-makers were from Antioquia; however, three of them worked at the national level and two at the local level. In the group of citizens, there was representation according to the residence (i.e., urban and rural areas), and socioeconomic status (i.e., strata one to five according to the Colombian system of classification). The educational level of those interviewed in this group varied from high school to postgraduate, eight reported having children and five reported belonging to a citizen organization.

### Perceptions about private insurance in Colombia

#### Phenomenon: VPHI is perceived as the solution to the lack of timely access to technologies and services

In Colombia, VPHI is perceived as a possible solution and as an opportunity to solve shortcomings in the public health system:

“Yes, it becomes a luxury, sadly, but the important thing is not whether there is someone with the ability to pay or not, nor whether someone sets up a private company in the health sector, there is nothing wrong with that. It is rather about correcting the shortcomings of the public sector, ensuring the guarantee of the right is the obligation of the State.” Administrator 01.

Some describe it as a “necessary evil”, considering it that should not exist if the public health system operated adequately. This is a quote from a healthcare worker:

“Precisely yesterday it happened with my mom, we saw barriers to access for one reason or another; fortunately, my brother pays for prepaid medicine. I said to my mother: “How is it possible that a health system like ours, with the resources we have… [we can] cover absolutely all the services,… [we have to] resort to prepaid, private medicine, to speed up the procedure?” Health personnel 09.

#### Context: Commodification of health and social stratification

The perceptions of the citizens regarding VPHI have been structured in the context of social stratification. In Colombia, homes are categorized on a scale from 1 to 6 according to their physical characteristics and the urban context in which they are located. According to this categorization, the government allocates subsidies to the lowest socioeconomic strata (categories 1 to 3). This social stratification policy has normalized the idea that people in higher strata have access to better public services, including healthcare. Regarding this topic, one participant expressed the following:

“Obviously, the stratification in a country like ours and in purely capitalistic countries will continue, and those with the possibility of paying and acquiring the diagnoses and treatment in a particular way will have it. However, the premise of all these studies is: Let’s try so that we can serve the entire population in a much more uniform, adequate, and fast manner, regardless of their socioeconomic status.” Health personnel 06.

As a result of the market-oriented reforms in 1993, the country has normalized that those with greater economic resources can access healthcare services with private funds. For some participants, this situation is not problematic, and they compare it with the fact that some citizens have access, depending on their ability to pay, to different luxury goods, such as certain brands of cars or five-star hotels:

“I do not want to mess with prepaid medicine because that is an out-of-pocket expense. That is: If I want to buy an Audi, why can’t I buy it if I have the money? Not everyone in the population of Colombia has an Audi. It is an out-of-pocket expense, and the prepaid medicine behaves this way; it is a way of marketing healthcare to gain access to rapid service.” Decision Maker 03.

However, other interviewees find it “unfair” that access to necessary health services is mediated by one’s ability to pay:

“Well, I believe that everyone should have the attention or the timely access to care that prepaid medicine offers. It seems unfair that one must pay for better care, it seems to me that it must be equalized, to be a service just as good for all the people and not have to pay additionally for having faster access to care.” Patient 02.

#### Causes: Lack of timely access to care in the public health system, extensive administrative procedures, and privileged attention-seeking

Citizens acquire VPHI due to the lack of timely access to care in the public system, extensive administrative procedures, and the search for privileged attention. Age is a factor in deciding to access private insurance, mainly due to concerns associated with timely access. The following quote illustrates this situation:

“Due to delays in the health system, it is when you reach a certain age that you access private health insurance, or for the beloved ones because you do not want them to suffer.” Citizen 14.

“Unfortunately, it is much more efficient because while you go to the public system and it takes 6 months for giving you an appointment, well, in a private system they give you [an appointment] immediately, obviously it is much more efficient.” Decision Maker 01.

The perceptions of the citizens reflect tension regarding the possibility of receiving privileged care. For some, it is fair that access to health services differs for individuals with different payment capabilities. It is even mentioned that VPHI is a way to relieve congestion in the public system. However, for others, timely access to healthcare and the quality of care must be the same for people who have different abilities to pay but have the same health needs.

“I think that our system would not hold if everyone were to be in the same coconut [referring to all populations in the public system] to access services, I think that it would collapse more than it is collapsed. Therefore, I think that it is valid that the system continues to have prepaid medicine.” Administrator 03.

“If I get cancer or whatever, I need to have timely access to be treated and they must offer me the same services and the same trained people as the one who has better resources than me.” Patient 05.

#### Consequences: lack of timely access to care in the public health system, double payment, and different outcomes for the same health needs

Some participants consider that the VPHI deteriorates the quality and increases the wait time for patients who use the public health system when providers are only allowed to work in the private system.

“However, if someone wants to pay, let them pay! However, there are some ethical minimums that should be guaranteed in the system because it is very paradoxical that they do not attend to you and there is no schedule for this specialist, but that same specialist does have a private schedule. No, well, that is an aberrational thing! These are ethical dimensions that have increasingly perverted the health system and the specialists.” Citizens 17.

Likewise, they recognize that having a VPHI in Colombia has the consequence of paying twice for the same benefits. In the Colombian health system, all health technologies that are necessary for the patient are covered and funded by the public system, except for explicit exclusions.

“In Colombia, it is not logical that there are complementary insurance plans, prepaid medicine, or private health insurance because all that is just paying twice and thrice for what you are already entitled to according to the law. I insist, denying or delaying services is the way to sell more prepaid medicine, more complementary plans, it is the insurance companies that play to denying the services to sell their prepaid medicine.” Health professional 07.

Other participants pointed out that the VPHI generates differences in healthcare that can spawn different health outcomes for patients who have the same care needs.

“What is being observed in the emergency department is quite intense. Everyone goes for a reason, so we do triage… the idea is that when you arrive, within ten minutes you should be told how long it will take, one hour, two hours, six hours. However, when they arrive with prepaid [medicine], they are triaged and immediately it’s like “oh, he pays more”,—the one who pays more is attended faster.” Health professional 08.

The combination of the lack of timely access to care in the public health system and the additional cost of purchasing duplicate private insurance has consequences for low-income individuals. To avoid paying a monthly premium for a VPHI, some people prefer to pay out-of-pocket for a private medical appointment, file a legal action, visit a drug store to access treatments without a medical prescription or, in extreme cases, avoid seeking medical consultation altogether and bear the negative consequences that this can have for their health. This is how one participant expressed it:

“That’s very sad, it’s like whoever wants better care simply pays more. In addition, [these private health plans] don’t have the most affordable fees, someone can say, great, I’m going to pay for this prepaid medicine! However, there are people who don’t have 400,000 pesos, which is what you normally pay; someone will say: that is what I make in a month!” Citizens 07.

#### Actions and interactions

There have been multiple actions from citizens regarding the existence of VPHI in Colombia. Some believe that it should not exist, while others defend it from a consumerist standpoint, stating “those who can afford it should pay for it,” or from its possibility of guaranteeing quality, timely access, and comfort in healthcare. This interaction within the health system has created a need for feeling safe in receiving timely care when needed, and this manufactured need is satisfied by buying timely access to care and safety in the market, which VPHI can provide. There is supply and there is demand. One participant stated:

“Well, it becomes that way because of the need that we have. You pay for prepaid medicine for your loved ones, parents, and children. Because you don’t want them to suffer from those situations of going to an emergency room or having to undergo interventions where one sometimes feels like begging.” Citizens 14.

Another interaction between the public health system and private health insurance is highlighted by the contradiction that arises when the demand for VPHI increases, leaving the public system gradually unprotected. This occurs when health professionals, in the exercise of liberal practice, provide their services exclusively in the private system and not in the public, worsening the shortage of human resources within the public system and the lack of timely access to care. In addition, the private health system feeds the idea that the solution to the problems of the public system can be solved through the purchase of VPHI and not with improvements to the public system. This quote reflects the above statement:

“It seems to me that the fact that prepaid medicine is allowed means that the health system does not make an effort to improve, that is, it continues to be bad. Because if people do not find in their EPS -public insurance company- the right wait times, nor the good treatments and medications, then they switch to the prepaid. That means that the public health sector does not improve; in contrast, it is worse because improvement is not a requirement of the health system. They are comfortable waiting for everything to be done by the prepaid, then I think it is counterproductive, there shouldn’t be prepaid medicine!” Patient 02.

## Discussion

Our study allowed us to explore the perceptions that Colombians have about VPHI. The paradigmatic matrix was relevant to organizing our understanding of how VPHI works in a context of health commodification and social stratification, the lack of timely access to care, extensive administrative procedures, and the pursuit of privileged care are perceived as the causes of why citizens acquire VPHI in Colombia. The perceptions about the consequences of the use of VPHI include the worsening of timely access to healthcare in the public system due to the recruitment of doctors and hospitals by the private sector; higher costs for citizens due to double payments for the technologies and services that all people are entitled to in principle; and the widening of inequity in access to healthcare services among individuals with similar needs but different payment capabilities.

In the Colombian context, we did not find publications with an approach such as ours. Four publications addressed the issue from a legal and jurisprudential perspective [23–25]. Three other articles approached it from an economic perspective with the interest of evaluating the sector as a business [26–28]. Despite the different approaches, four publications highlight that private health insurance emerges as a response to problems of timely access to health services [23, 24, 26, 28]. One publication concludes that in the VPHI, the insurer commits through a contract to provide a service of “hospitality and luxury” [24]. However, it is important to emphasize that ensuring better timely access to healthcare is not a “luxury” and that limitations to healthcare access reflect the inequity of the system since citizens with the greatest ability to pay have access to care when asked, with possible negative implications for other citizens with the same or greater need for care but with less ability to pay.

In the Latin American context, other authors have also reported on the possible inequality implications of VPHI. In Brazil, where duplicate VPHI exist, individuals who are double covered by the public system and private insurance, which are the ones with better socioeconomic status, have the possibility to use the resources from the public system when their plans do not cover the type of service required. According to these authors, this dual use of resources by individuals with VPHI has the potential to increase health inequalities and restrict universality [29–31]. This tension between the VPHI and the public system is present in other Latin American countries. In Argentina, there are disparities in access, use, and quality of healthcare between individuals who have access to private insurance, which are generally the ones with the highest income, and the ones that are covered by the social security system [32]. In Chile and Uruguay, the difference in the quality of health care between the public systems and the private systems keeps pushing higher-income individuals into private insurance [33].

### Strengths and limitations

We identified the strengths and limitations of this study. The main strength lies in the possibility of integrating visions, opinions, and experiences of a diverse group of citizens. From the planning stage, we looked for a heterogeneous group of participants who brought different voices according to sex, age, educational level, place of residence, knowledge and experience with the health system, socioeconomic condition, affiliation with the social security health system and experience in the use of voluntary private health insurance. We consider that the diversity of perspectives enriched the analysis and allowed for a more holistic understanding of the phenomenon. Although, the design chosen for this study could be considered a limitation due to its qualitative nature; the choice of design was intentional since we sought to understand connections, interactions, perceptions, and experiences about VPHI, and this understanding allowed us to propose a conceptual framework that explore them, we did not pretend to make generalizations about what most citizens think about voluntary private insurance.

## Conclusion

This qualitative analysis allowed us to better understand citizens' rationale for purchasing VPHI and their perceptions about the possible implications of worsening equity gaps in the Colombian health system. This can be helpful information for policymakers who are designing policies about the role of the private sector in healthcare, especially in the access to services that are necessary for the health and well-being of the entire population. For citizens, this research shows how VPHI is perceived as a double payment for services covered within social security plans and suggests that the lack of timely access to care in the public systems and the fear that citizens have for themselves or their family members when using suboptimal healthcare at some point may be used by VPHI companies to encourage the purchase of these private insurances. Finally, it is important to highlight that our study was designed to explore the perceptions of Colombians about VPHI and that other types of studies are more suitable to assess the effects of VPHI on health equity in Colombia. We consider it relevant to conduct further research to determine, for example, the impact of VPHI on the availability of resources in the public system, including the impact on the opportunity of care for those that do not have private insurance.

### Supplementary Information

**Additional file 1.**

## Data Availability

Availability of audio recordings and interview transcriptions.

## Abbreviations

VPHI: Voluntary Private Health Insurance.
EPS: Intermediate insurance companies (for its acronym in Spanish).
IPS: Health care institutions (for its acronym in Spanish).
CD: Citizens.
PC: Patient.
TD: Decision Maker.
PS: Health personnel.
AD: Administrator
