# Supplementary material for: Whoever wants better healthcare simply pays more: citizens' perception about voluntary private health insurance in Colombia
Source: Int J Equity Health. 2024 Jan 12;23:7. doi: 10.1186/s12939-023-02086-z (PMC10785507; doi:10.1186/s12939-023-02086-z)
Supplement: Supplementary file 1 — Additional file 1. [file 12939_2023_2086_MOESM1_ESM.docx]

**Appendix**

The qualitative information presented in this article comes from a series of interviews we conducted for a project entitled "Citizen perceptions about technologies and services to be covered with public resources of the health system, and the role of evidence in their modification". In the question guide for this project, we included a question on perceptions about voluntary private health insurance plans (VPHI) also called prepaid medicine in Colombia, and an example to illustrate the topic. Below, we present the entire interview guide, but the article refers mainly to the analysis we made of question number 4 and the example presented in situation number 5.

| **Semi structured interview question guide**  **-Version 4-** |
| --- |

| **I. Home** |
| --- |

*Initial greeting.*

*Introduction about who we are and the objective of the activity: We are a group of people, researchers from the Faculty of Medicine of the University of Antioquia, who are conducting a study in which we would like to know your opinion about the things in health that you think should or should not be provided to people without having to pay extra money out of pocket or pay for it privately.*

*We call these "things in health" technologies, and by that word, we mean drugs, medical consultations, surgeries, laboratory tests or radiology imaging, vaccines, and devices, i.e., everything that serves to make a diagnosis, to treat a disease or condition, and/or for health rehabilitation.*

*Clarifications on the voluntary nature of participation: If there is any question that you do not want to answer or if you wish to stop the interview, there is no problem, you can calmly indicate it to the interviewer.*

*You can answer calmly because this interview is confidential. If you accept, we will record it, and when it is finished, it will be transcribed. When it is transcribed, we will remove your name from it, and with this written interview, we will conduct the analysis for the research. We appreciate your space and availability for this meeting.*

**It is necessary to confirm that the interviewee has approximately one hour to develop the interview or two hours minutes for the focus group participation.*

| ***If it is a one-on-one interview:***  ***- Is it okay if we start the recording?***  ***- Can you tell me your position (occupation)?***  ***- How long have you been in this position (occupation)?*** | ***If it is a focus group:***   - ***Is it okay if we start the recording?*** - ***- Each of the members is kindly asked to indicate his/her name, occupation (position) and how long he/she has been in that position (or occupation).*** |
| --- | --- |

| **II. Interactive communicative process: elaboration of questions** |
| --- |

| 1. What do you think should be covered or paid for with health system resources? Why?   **Prompts (we must identify if what is mentioned is paid with UPC or by Mipres)** |
| --- |

- - All/some things/most things
  - Depends on the person's ability to pay
  - Depends on patient age
  - Depends on the disease
  - Depends on how expensive the technology is
  - Depends on how effective the technology is
  - Whatever the doctor orders/whatever the Constitutional Court says/whatever is defined in a benefits plan
  - Affected by factors such as corruption, distrust in IPS or EPS, lack of participation in decisions on inclusion/exclusion, other

| 1. ¿In your personal or family experience, do you remember any technology that you have paid for privately and that you consider should be covered by the health system (medications, surgeries, medical exams, consultation with specialists, other therapies such as naturopathic ones)? Why?   **Prompts (we must identify if what is mentioned is paid with UPC or by Mipres)** |
| --- |

- Ordered by physician/guardian
- Effective
- Costly
- Fair/dignified/equitable/equitable/for a vulnerable group

| 1. Which health technologies do you consider that people should pay for privately and that should not be financed with resources from the health system? Why?   **Prompts (we must identify if what is mentioned is paid with UPC or by Mipres)** |
| --- |

- None/some/most of the things

- Depends on the patient's ability to pay

- Depends on the patient's age

- Depends on the disease

- Depends on the cost of the technology

- Depends on how effective the technology is

- Anything not ordered by the physician/what is denied under conservatorship/what is not included in a benefit plan

- Cosmetic, experimental, offered outside the country, not proven to be effective, not approved for use.

- Affected by factors such as corruption, distrust in IPS or EPS, lack of participation in decisions on inclusion/exclusion, other

| 1. ¿What do you think about people who have prepaid medicine or who can pay privately, having faster access to health technologies?   **Prompts** |
| --- |

- To receive faster attention in emergency services.

- Faster appointments with specialists.

- Have certain procedures performed faster.

| **Discussion of perceptions on specific aspects** |
| --- |

Briefly, I am going to give you some examples of situations that arise in the health system, and then I will ask you whether you believe that, in such a situation, the technology should be paid for with public or private resources.

| **Situation 1** |
| --- |

**"The mother of a 12-year-old boy with cerebral palsy filed a tutela against EPS X, after it refused to deliver 180 diapers and three packages of wet wipes for a period of three months, among other requirements made by a hospital in Yopal. The EPS X granted the requested medicines but denied the coverage of the diapers and wet wipes, arguing that they were items that were not covered by the Health Benefits Plan". (El Espectador March 23, 2018)**

1. **¿Considera 5. Do you think the health system should cover diapers for this child?**
2. **Do you think that diapers should be provided by the health system to all people who require them, or should they be purchased at the families' own expense? Why?**

**Prompts**: Require additional information, some factor affecting the decision (patient's ability to pay, age, disease, frequency of disease, how costly, how effective, who ordered it).

| **Situation 2** |
| --- |

**The father of a pair of 10-month-old twins who suffer from a very rare disease (Spinal Muscular Atrophy), which only 70 people in Colombia have, filed a tutela requesting that the pharmacological treatment for his daughters (Spinarza) be covered. The treatment has few studies that properly evaluate it, and those that exist show that it can, in 1 out of every two children, allow them to carry out some movements (raising their arms or head control, for example), but it does not change the course of the disease. This treatment costs $551 million pesos per injection, and one year's treatment for the two girls would cost $7,346 million pesos**^[[1]](#footnote-1)^**.**

1. **Do you think that the health system should cover the twins' medication?**
2. **Do you think that highly expensive drugs should be covered by the public resources of the health system? Why?**

**Prompts**: Require additional information, some factor affecting the decision (patient's ability to pay, age, disease, frequency of disease, how costly, how effective, who ordered it).

| **Situation 3** |
| --- |

**A 59-year-old woman underwent breast reconstructive surgery after suffering from cancer. EPS X denied her surgery, claiming that the reconstruction of her breast was a cosmetic surgery, which had no effect on the treatment of her disease. (El Tiempo, February 20, 2019)**

1. **Do you consider that the health system should cover breast surgery for women?**
2. **Do you think that cosmetic breast augmentation surgeries should be covered by the public resources of the health system? Why?**

**Prompts**: Require additional information, some factor affecting the decision (patient's ability to pay, age, disease, frequency of disease, how costly, how effective, who ordered it).

| **Situation 4** |
| --- |

**"In Colombia, when we talk about "alternative therapies", we refer to techniques and practices other than conventional medicine. That is, this group includes acupuncture, aromatherapy, oriental medicine practices or homeopathic medicine. However, centers that do aba, equine therapy, dog-assisted therapy, dolphin therapy, among others, are NOT considered alternative therapies." (Eliech Comment, July 4, 2013:** [**http://agaviria.co/2013/06/terapias-aba-otro-fraude-al-sistema-de.html**](http://agaviria.co/2013/06/terapias-aba-otro-fraude-al-sistema-de.html)**).**

1. **Do you consider that these types of therapy should be covered by the public resources of the health system? Why?**

**Prompts**: Require additional information, some factor affecting the decision (depends on technology, patient's ability to pay, age, disease, frequency of disease, how costly, how effective, who orders it).

| **Situation 5** |
| --- |

**A 45-year-old woman was diagnosed with a pelvic mass. To clarify its origin and characteristics, an ultrasound was ordered, but her EPS only had an appointment for two months later; however, as the patient had Prepaid Medicine, she resorted to it, and the ultrasound was performed two days later. The result showed a mass in the right ovary; surgery was performed, and the biopsy showed a malignant ovarian tumor, which led to immediate treatment.**

1. **What do you think about the use of prepaid medicine in this case?**
2. **Do you consider it appropriate that in Colombia, those who have resources can have prepaid medicine (or complementary plans) and have more timely care and other benefits? Why?**

**Prompts**: Require additional information, some factor affecting the decision (age, disease, frequency of disease, cost, effectiveness, who orders it).

| **III. Closing** |
| --- |

We thank them for their time and willingness to participate in this space, reiterate the importance of their participation in the project and additionally ask the following questions to close the activity:

- **Is there anything else you would like to add about the above?**
- **Do you have any questions to ask me?**
- **Do you have any concerns before I stop the recording?**

Thank you for answering these questions and sharing your view.

Appendix table 1. Participant characteristics by group.

|  | **Citizens (N=18)** | | | **Patients (N=5)** | | | **Professionals (N=13)** | | | **Administrators (N=5)** | | | **Decision Makers (N=5)** | | |
| --- | --- | --- | --- | --- | --- | --- | --- | --- | --- | --- | --- | --- | --- | --- | --- |
| Age range |  | 29-66 | |  | 25-59 | |  | 23-57 | |  | 31-46 | |  | 37-55 | |
|  |  | **n** | **%** |  | **n** | **%** |  | **n** | **%** |  | **n** | **%** |  | **n** | **%** |
| **Women** |  | 11 | 61.1 |  | 4 | 80,0 |  | 7 | 53.8 |  | 2 | 40,0 |  | 2 | 40,0 |
| **Health insurance scheme** |  |  |  |  |  |  |  |  |  |  |  |  |  |  |  |
| Subsidized |  | 3 | 16,7 |  | 2 | 40,0 |  |  |  |  |  |  |  |  |  |
| Contributory |  | 11 | 61,1 |  | 2 | 40,0 |  | 13 | 100,0 |  | 3 | 60,0 |  | 5 | 100,0 |
| Especial |  | 1 | 5,6 |  | 0 | 0 |  |  |  |  |  |  |  |  |  |
| VPHI |  | 3 | 16,7 |  | 1 | 20,0 |  | 2 | 15,4 |  | 2 | 20,0 |  | 1 | 20,0 |
| **Municipality of residence** | Medellín | 8 | 44,4 | Medellín | 2 | 40,0 | Bucaramanga | 4 | 30,8 | Rionegro | 2 | 40,0 | Medellín | 4 | 80,0 |
|  | Ebéjico | 2 | 11,1 | Bello | 1 | 20,0 | Bogotá | 2 | 15,4 | Ebéjico | 1 | 20,0 | Marinilla | 1 | 20,0 |
|  | San Jerónimo | 2 | 11,1 | Bucaramanga | 1 | 20,0 | Medellín | 6 | 46,2 | Medellín | 1 | 20,0 |  |  |  |
|  | Bello | 1 | 5,6 | Villavicencio | 1 | 20,0 | Yolombó | 1 | 7,7 | Mutatá | 1 | 20,0 |  |  |  |
|  | Manizales | 1 | 5,6 |  |  |  |  |  |  |  |  |  |  |  |  |
|  | Envigado | 1 | 5,6 |  |  |  |  |  |  |  |  |  |  |  |  |
|  | Bogotá | 3 | 16,7 |  |  |  |  |  |  |  |  |  |  |  |  |
| **Group-specific variables** | **Place of residence** |  |  | **Site of service provision** |  |  | **Sector** |  |  | **Level of administration** | |  | **Reach of decisions** | |  |
|  | Urban | 14 | 77,8 | Hospital care | 1 | 20,0 | Public | 7 | 53,8 | IPS | 3 | 60,0 | Local | 2 | 40,0 |
|  | Rural | 4 | 22,2 | Outpatient care | 4 | 80,0 | Private | 6 | 46,2 | Health secretariat | 2 | 40,0 | National | 3 | 60,0 |
|  | **Socioeconomic strata** | |  | **Disease or diagnostic** | |  | **Site of service provision** |  |  |  |  |  | **Institution** |  |  |
|  | One | 3 | 16,7 | Infection | 1 | 20,0 | Consult | 5 | 38,5 |  |  |  | Private | 3 | 60,0 |
|  | Two | 3 | 16,7 | Chronic | 1 | 20,0 | Hospitalization | 3 | 23,1 |  |  |  | Public | 2 | 40,0 |
|  | Three | 3 | 16,7 | Transit accident | 1 | 20,0 | Emergency unit | 4 | 30,8 |  |  |  |  |  |  |
|  | Four | 7 | 38,9 | Pediatrics | 1 | 20,0 | Pharmacology and toxicology | 1 | 7,7 |  |  |  |  |  |  |
|  | Five | 2 | 11,1 | Cancer | 1 | 20,0 |  |  |  |  |  |  |  |  |  |
|  | **Level of education** |  |  |  | | | | | | | | | | | |
|  | High school | 3 | 16,7 |  |  |  |  |  |  |  |  |  |  |  |  |
|  | Technical level | 3 | 16,7 |  |  |  |  |  |  |  |  |  |  |  |  |
|  | Undergraduate | 6 | 33,3 |  |  |  |  |  |  |  |  |  |  |  |  |
|  | Graduate | 6 | 33,3 |  |  |  |  |  |  |  |  |  |  |  |  |
|  | **Children** |  |  |  |  |  |  |  |  |  |  |  |  |  |  |
|  | Yes | 8 | 44,4 |  |  |  |  |  |  |  |  |  |  |  |  |
|  | **Belongs to citizen organization** | | |  |  |  |  |  |  |  |  |  |  |  |  |
|  | Yes | 5 | 27,8 |  |  |  |  |  |  |  |  |  |  |  |  |

Appendix table 2. Participants quotes organized according to the paradigmatic matrix.

Relevant phrases in the Patients group

| Context | Causes | Consequences | Actions & Interactions |
| --- | --- | --- | --- |
| That's a big problem and that's capitalism at 100 percent... So that's not only in health, in housing, in food, it's in everything, – those who have money have better access to things than those who don't have money – whether it's fair or not, possibly it isn't but it's the reality in which we live, suddenly that they don't have the same level. but that they can survive. Suddenly, those who don't have money are attended to in an hour and those who have are attended to in ten minutes, because that can be it; The one who has money has better access to things than the one who doesn't, in everything... Not only in health. (PCTR-PC5)  – I say that it depends, for example: "On the assumption that we all have EPS and prepaid medicine does not exist" So I have more money and I want to pay more therapy for my son or for my family, and if I am going to pay more than what medicine for everyone really covers, I don't see a problem because everyone has their own resources. It's like high-performance athletes who pay for more training, more specialists and the whole story, but that it covers the minimum is the quality of life that I'm telling you. (PCTR-PC5)  – If the EPS does it for me after three months and the complementary plan does it for me the next day, I need to know if that mass is already malignant and not put up with it for three months; if we see it on paper it shouldn't be, but if we focus on reality, in this system if one can pay, welcome to those who can pay and we leave the time of the EPS to those who suddenly need it faster through the EPS. (PCTR-PC04.)  "But of course it's good!" At that time... Actually, the ones he gave me have, we have like this, how to pay or how to pay for a prepaid medicine or how to pay for some tests or how to pay for the appointment, we are blessed because there are people who do not have that possibility and should have it and should have it... The health system should be prepared for that. (PCTR_PC03)  "Well, that's right, exactly, exactly, I mean, because there are people, right?" What we are saying as a hotel, that is, there are people who have the means to go and pay for a five-star hotel and go for a five-star hotel, that's how I say, since there is prepaid medicine for people who pay to be better, well, man! It has money and they want it, they want to use it for that... I don't disagree with that, I think I agree, I don't pay it but there will be a lot of people who pay it, right? (PCTR_PC03)  "It's very sad, but it's true..." From my point of view, thank God I am a person who can have access to prepaid medicine, some of my relatives too, but looking at it from the point of view of people who live in villages, a person goes to ask for an appointment from a town and they tell him that he will stop tomorrow and he no longer has the tickets for that while another person who has prepaid medicine goes any day and even for a short time. phone number gives it to you; That seems a bit unfair to me because the people who live in the villages are really too needy and they don't have that quality of life that one can give, so I think that's a very big injustice, because it's very sad but yes. (PTCR-PS12)  – There should be prepaid medicine, so I say for people who like to be well taken care of, like when there is a hotel, there are two-star, three-star, four-star, five-star hotels, right? then if prepaid medicine should exist for people who like five-star medicine (PCTR-PC03)  "Or a young man who earns five million and nothing happens to him and pays more..." It's like a pension-like structure. (PCTR-PC5) | –I think that if I pay for a prepaid or policy to access a faster service, it is valid, (PCTR-PC04)  "For my sake, that's what I'm telling you if you're going to have prepaid medicine to access a service faster, I totally agree. (PCTR-PC04)  "And we all start from the same point, do you know what I mean?" If I get cancer or whatever, I have to have the opportunity to be treated and I should be offered the same service and the same trained people as those who have better resources than me, (PCTR-PC5) | "I say—" I was even telling him that I used to pay for the supplemental plan. The complementary plan was like to speed up, speed up certain appointments, but I didn't notice much difference either... let's say this there is and no, I didn't think so because the convenience didn't satisfy me ( PCTR-PC03)  "I think—" that the prepaid system for people who are in a position to pay it, who are in those conditions because they can continue to do so (PCTR-PC03)  "Look, it's not fair, I'm going to tell you something..." with a colleague, she needed an urgent exam, ready, by the EPS I called and called and called and there was nothing... There were no appointments and he called a... for the prepaid, for the prepaid and there were appointments, well! (lifts shoulders) (PCTR_PC01)  In other words, that's something that the EPS are playing for, they stop... They stop serving people for... to people who don't have resources for people who are paying more. (PCTR_PC01) | – I understand that even when it is a technology sent by prepaid where it is the prepaid that orders but with the EPS pocket, then the specialist ordered it but they charge it to the EPS; That should have a validation, we all know that the disability of the headache for the patient of the policy is not the same as the disability for the patient of the POS, but the disability is paid by the EPS, so that is where one begins to differentiate, (PCTR-PC04) |

Relevant phrases in the Healthcare Workers group

| Context | Causes | Consequences | Actions and interactions |
| --- | --- | --- | --- |
| "But it is understood that due to the overflow and the inability to attend to the entire population, it makes it stratified, I have experienced that very frequently and specifically with my parents – To whom I do wait for my mother to have an MRI on her knee so that I can look for the possibility of a knee prosthesis, Well, they die waiting for the prosthesis, in fact that's what happens very often, so that's where I think we should give them the resources to try to get everyone to be in a similar trap. (PCTR-PS06)  – I said it in the previous question, right or wrong, that will continue to exist in a country like ours and in capitalist countries where socio-economic strata exist. (PCTR-PS06).  Obviously, the stratification in a country like ours and in purely capitalist countries will continue, and those who have the possibility of paying and acquiring the diagnosis and treatment in a particular way will have it. But the premise of all these studies is: – Let's try to treat the entire population in a much more uniform, adequate and rapid way possible regardless of their socioeconomic status. (PCTR-PS06).  "Oh well, tasty... Every man for himself" and there should be no prepaid medicine, we have to end that business of the health market.  I don't totally agree and just yesterday it happened to me with my mother, fortunately my brother has my mother in a category of prepaid medicine and we saw barriers to access on the one hand and on the other hand I said to my mother: "How is it possible that in a health system like ours with resources that we have but that are diverted by the mercantilist model that we have; That the same system has enough resources could cover absolutely all services and we would not have to resort to a prepaid, private medicine to speed up the procedure. PCTRPS09  In other words, I am not against prepaid medicine, but looking at it from the other side for those who are not there, it is unfair that it is prioritized for an economic issue rather than for the urgency that the patient has to make his diagnosis and thus his timely treatmentPCTR PS10  "So I don't agree with the existence of prepaid medicine; That should be eliminated, we're filling up his coffers... PCTRPS09  Q2: Again, I say that early testing should be better for everyone. It is not fair or equitable that the person has to pay a little more to get an exam in less time. The health system in Colombia should commit to that and ensure that studies are at the same time for all people. Obviously in Colombia the health system has many problems and one of them is that waiting time for paraphiliacs, in that case one as a person assumes the cost, but it should not be money from the pocket of each one of us but from the government itself, it should guarantee that we all have the same health conditions. (PCTR-PS02)  – Well, I think we should all have the attention or urgency that a prepaid medicine offers, it seems unfair to me that one has to pay for a better service, it seems to me that it has to be equalized, the POS service has to be equally good for all people and not have to pay extra for having less time. I think that the health system is going to collapse from now on in terms of organizing itself in terms of timing, but it seems unfair to me that you have to pay more for something that you have to pay for from the POS service. (PCTR-PR02). | –That is; It's absurd, the opportunity should be for everyone, in that sense what they're looking for is to create need, it's to create more, pay me more and I'll attend to it faster. PCTRPS08  – But notice that the problem is not the prepaid medicine, it is not the alternative services that are bad, this one is there but because the others do not supply, then they exist. The reflection would be: Why wouldn't others be able to provide that attention? That's what I would believe. (PCTR-PS05)  "I'd think not, in terms of timely care. I think that is the problem of the health system as such, because otherwise we would be making a differential in the provision of services, where those who have resources then have more possibilities of being treated faster or being cured of cancer. (PCTR-PS05). | It seems to me that the fact that prepaid medicine is allowed means that the health system does not make an effort to improve, that is, that it continues to be bad, because the person does that. He does not find in his EPS the times or the good treatments and medications; then it goes to the prepaid; that makes the EPS; the health system is comfortable with that and people are increasingly turning to prepaid medicine, so that does not improve the health issue, on the contrary it is worse, because the health system is not required to improve; They are comfortable waiting for everything to be done by the prepaid, so it seems counterproductive to me, there should be no such thing as prepaid! (PCTR-PS02)  – I can't conceive of these companies that are filling their coffers, to provide the same services that we pay for contributors, and for the same services of the subsidized regime. PCTRPS09  –Terrible! "He who has the money has the power" Nope! In that part, I try to be very equitable. What are you seeing in the emergency department that is very strong?: Everyone goes for a reason, so we do triage, it takes ten minutes, the idea is that when you arrive, in ten minutes you should be told how long it takes, an hour, two hours, six hours, that it can wait and it is contemplated. But when he arrives with prepaid, he is triaged and it is done once and for all "ah is that he pays more", – the one who pays more is attended faster – totally absurd! PCTRPS08  Well, I think we should all have the attention or urgency that a prepaid medicine offers, it seems unfair to me that one has to pay for a better service, it seems to me that it has to be equalized, the POS service has to be equally good for all people and not have to pay extra for having less time. I think that the health system is going to collapse from now on in terms of organizing itself in terms of timing, but it seems unfair to me that you have to pay more for something that you have to pay for from the POS service. (PCTR-PR02).  "My mom is lucky, she's had prepaid medicine all her life and when she needs surgeries or procedures it's been like this [snaps fingers] In four days. But how many Colombians are waiting for access to a health technology through their regime, including in the contributory regime? PCTPS09 | I believe that the problem there would not be prepaid medicine, but the inability of the health system to adequately redirect the patient. The fight shouldn't be with prepaid medicine. That example shows what it shows at the same time: is that there can be certain types of contracting of an EPS with certain institutions, sometimes that limitation in appointments or imaging studies is given because they concentrate their contracts in one entity and that generates that the needs of the population they have affiliated cannot be met. It is that if it worked differently and not only with whom I contract the services specifically, those problems could be overcome, just as it is illogical that one has to travel from one end of the city to the other to be treated, really the problem lies in the fact that the contract of your EPS is with another clinic, so you have to move there, knowing that there are other institutions that can serve you closer. That shows the problem of the health system to be decisive in this case. (PCTR-PS05).  I think there is a bad intention on the part of the EPS, that person needed to have that procedure done within their PBS, therefore there should have been no barrier to access to the service. Because the doctor was really there and was able to see him two days later. In other words, what the EPS did was to generate an administrative barrier to access to health services, that should not only be rejected, but punished. (PS-07)  There should be no prepaid medicine because the system has to work efficiently and there has to be a restructuring. PCTRPS09 |

Relevant phrases in the Healthcare Workers group

| Context | Causes | Consequences | Actions and interactions |
| --- | --- | --- | --- |
| "But it is understood that due to the overflow and the inability to attend to the entire population, it makes it stratified, I have experienced that very frequently and specifically with my parents – To whom I do wait for my mother to have an MRI on her knee so that I can look for the possibility of a knee prosthesis, Well, they die waiting for the prosthesis, in fact that's what happens very often, so that's where I think we should give them the resources to try to get everyone to be in a similar trap. (PCTR-PS06)  – I said it in the previous question, right or wrong, that will continue to exist in a country like ours and in capitalist countries where socio-economic strata exist. (PCTR-PS06).  Obviously, the stratification in a country like ours and in purely capitalist countries will continue, and those who have the possibility of paying and acquiring the diagnosis and treatment in a particular way will have it. But the premise of all these studies is: – Let's try to treat the entire population in a much more uniform, adequate and rapid way possible regardless of their socioeconomic status. (PCTR-PS06).  "Oh well, tasty... Every man for himself" and there should be no prepaid medicine, we have to end that business of the health market.  I don't totally agree and just yesterday it happened to me with my mother, fortunately my brother has my mother in a category of prepaid medicine and we saw barriers to access on the one hand and on the other hand I said to my mother: "How is it possible that in a health system like ours with resources that we have but that are diverted by the mercantilist model that we have; That the same system has enough resources could cover absolutely all services and we would not have to resort to a prepaid, private medicine to speed up the procedure. PCTRPS09  In other words, I am not against prepaid medicine, but looking at it from the other side for those who are not there, it is unfair that it is prioritized for an economic issue rather than for the urgency that the patient has to make his diagnosis and thus his timely treatmentPCTR PS10  "So I don't agree with the existence of prepaid medicine; That should be eliminated, we're filling up his coffers... PCTRPS09  Q2: Again, I say that early testing should be better for everyone. It is not fair or equitable that the person has to pay a little more to get an exam in less time. The health system in Colombia should commit to that and ensure that studies are at the same time for all people. Obviously in Colombia the health system has many problems and one of them is that waiting time for paraphiliacs, in that case one as a person assumes the cost, but it should not be money from the pocket of each one of us but from the government itself, it should guarantee that we all have the same health conditions. (PCTR-PS02)  – Well, I think we should all have the attention or urgency that a prepaid medicine offers, it seems unfair to me that one has to pay for a better service, it seems to me that it has to be equalized, the POS service has to be equally good for all people and not have to pay extra for having less time. I think that the health system is going to collapse from now on in terms of organizing itself in terms of timing, but it seems unfair to me that you have to pay more for something that you have to pay for from the POS service. (PCTR-PR02). | –That is; It's absurd, the opportunity should be for everyone, in that sense what they're looking for is to create need, it's to create more, pay me more and I'll attend to it faster. PCTRPS08  – But notice that the problem is not the prepaid medicine, it is not the alternative services that are bad, this one is there but because the others do not supply, then they exist. The reflection would be: Why wouldn't others be able to provide that attention? That's what I would believe. (PCTR-PS05)  "I'd think not, in terms of timely care. I think that is the problem of the health system as such, because otherwise we would be making a differential in the provision of services, where those who have resources then have more possibilities of being treated faster or being cured of cancer. (PCTR-PS05). | It seems to me that the fact that prepaid medicine is allowed means that the health system does not make an effort to improve, that is, that it continues to be bad, because the person does that. He does not find in his EPS the times or the good treatments and medications; then it goes to the prepaid; that makes the EPS; the health system is comfortable with that and people are increasingly turning to prepaid medicine, so that does not improve the health issue, on the contrary it is worse, because the health system is not required to improve; They are comfortable waiting for everything to be done by the prepaid, so it seems counterproductive to me, there should be no such thing as prepaid! (PCTR-PS02)  – I can't conceive of these companies that are filling their coffers, to provide the same services that we pay for contributors, and for the same services of the subsidized regime. PCTRPS09  –Terrible! "He who has the money has the power" Nope! In that part, I try to be very equitable. What are you seeing in the emergency department that is very strong?: Everyone goes for a reason, so we do triage, it takes ten minutes, the idea is that when you arrive, in ten minutes you should be told how long it takes, an hour, two hours, six hours, that it can wait and it is contemplated. But when he arrives with prepaid, he is triaged and it is done once and for all "ah is that he pays more", – the one who pays more is attended faster – totally absurd! PCTRPS08  Well, I think we should all have the attention or urgency that a prepaid medicine offers, it seems unfair to me that one has to pay for a better service, it seems to me that it has to be equalized, the POS service has to be equally good for all people and not have to pay extra for having less time. I think that the health system is going to collapse from now on in terms of organizing itself in terms of timing, but it seems unfair to me that you have to pay more for something that you have to pay for from the POS service. (PCTR-PR02).  "My mom is lucky, she's had prepaid medicine all her life and when she needs surgeries or procedures it's been like this [snaps fingers] In four days. But how many Colombians are waiting for access to a health technology through their regime, including in the contributory regime? PCTPS09 | I believe that the problem there would not be prepaid medicine, but the inability of the health system to adequately redirect the patient. The fight shouldn't be with prepaid medicine. That example shows what it shows at the same time: is that there can be certain types of contracting of an EPS with certain institutions, sometimes that limitation in appointments or imaging studies is given because they concentrate their contracts in one entity and that generates that the needs of the population they have affiliated cannot be met. It is that if it worked differently and not only with whom I contract the services specifically, those problems could be overcome, just as it is illogical that one has to travel from one end of the city to the other to be treated, really the problem lies in the fact that the contract of your EPS is with another clinic, so you have to move there, knowing that there are other institutions that can serve you closer. That shows the problem of the health system to be decisive in this case. (PCTR-PS05).  I think there is a bad intention on the part of the EPS, that person needed to have that procedure done within their PBS, therefore there should have been no barrier to access to the service. Because the doctor was really there and was able to see him two days later. In other words, what the EPS did was to generate an administrative barrier to access to health services, that should not only be rejected, but punished. (PS-07)  There should be no prepaid medicine because the system has to work efficiently and there has to be a restructuring. PCTRPS09 |

Relevant phrases in the decision-making group

| Context | Causes | Consequences | Actions & Interactions |
| --- | --- | --- | --- |
| - "One like that feeling of sadness that users who arrive at the Ministry of Health tell you because they have been 4-5 months ago, 6 months behind an appointment with a hemodynamic cardiologist or with an ehh neurologist who I don't know what, they get the appointment, they go and in five minutes they are dispatched then..."-and what did he tell you but they arrived at...? "No, no, he didn't tell me, I didn't, he could have said it over the phone over a video call," right?... And, and it's that patient who is waiting for and who turns out to arrive and look at the story in 5 minutes and say "no, this is not mine, that's up to the, I don't know what, of the, of the last subspecialty that is in the country or that is in... or that he only has three specialists in Medellín and that they line up for a year to be seen in, in, in those 15 minutes." AD02 - "I don't want to mess with prepaid medicine because that's an out-of-pocket expense. That is to say: "If I want to buy an Audi®, why can't I buy it if I have money?" And not everyone in Colombia owns an Audi®. It's an out-of-pocket expense and prepaid medicine behaves that way, it's a way of commercializing to turn the health business into having quick access. What I would say is that the Colombian population has more and more health coverage, that is, we have a coverage that is around 94% in studies last year by the faculty of economics of the University of Antioquia that is managed by Dr. Humberto." TD03 - "That's like a private payment, we can't mix the public with the private, there are people who have private clinics and access is private; I would no longer be immersed in the health sector as such, I believe that it is not necessary to differentiate whether it is good or bad; I think that if someone can set up a private clinic and have something particular and if there are people who can access that, then wonderful, and if it becomes a luxury, sadly, but the important thing is not whether there is someone who has the ability to pay or not or that someone sets up a private company in the health sector. There is nothing wrong with that, it is more necessary to correct the shortcomings from the public sphere, in the guarantee of the right that is an obligation of the State." AD01 | - "The ones who have what, right? they are accessing health services in a more timely manner because there is... I mean, there's discrimination, I mean, poverty discriminates, poverty creates barriers and... I think it shouldn't be like that, that is, the, the, the pathologies should be prioritized but sometimes I also see that there is a kind of management, also economic, of our health system... where they also give you the la, la, to convince you to buy a plan or a prepaid medicine that are always talking about opportunity" AD02 - "Yes, we have done it, that is, not only with oneself but with the family group, what we haven't... Suddenly, in some things that tell you are covered or comes back from, from not being covered a certain procedure to carry, waiting for an authorization, a signature then... All that list that you made me at some point I have done either because of denial of the system or to gain agility in the process of solving the problem itself, not having to wait because the thing is urgent, put it that it is not life or death but yes, we have done it or go to some therapy leaving Western medicine, of our medicine, looking for other alternatives in the face of the no, in the face of not obtaining the results, then one goes to other types of therapies, medications, tests that one pays for, as I say, to speed up the process." AD03 - "The one who pays for prepaid medicine is paying for opportunity, the coverage one would say is the same and that's why for so long I opposed Sandra telling me "the prepaid, the prepaid" and I said "it's paying twice for the same thing, we have acquaintances, we are in the middle, we can move our influences, our friendships" so... logically, the one who is paying is paying for the opportunity and one has experienced it, what happened with Ana María, so eehhhh, it seems to me that the system is well used" AD03 - "That's an unreal world eehhhh I think our system doesn't, it wouldn't stand for everyone to be in the same way, in the same bogeyman to access services, I think it would collapse more than it is collapsed. So I think it's valid that the system continues to have the medicine part of prepaid medicine." AD03 - "I would quickly say as if to say: 'That's what we pay for, right?' I even say it from my own experience, when you take a plan of this type it's basically for that, right? Even one of the things that encourages one to have one of those policies. It's basically that, direct access and being able to access where you want and faster." TD 02 | - "Very good, if luxury exists, it is for those who can acquire it and wonderful; but we as a State is where we must guarantee the right and that is where, compared to the private sector, we are going to be much more lame and with the lack of opportunity that ultimately impacts with the complication of the patient in the future and obviously greater cost for the State because we neither detect nor attend in time, then, of course, the complication, even for the State, is much more costly, for the patient it is a risk and the shortcoming, as I said initially: Not guaranteeing the right to life or health; regardless of whether there are others who can deliver luxuries or not." AD01 - "The issue of prepaid goes beyond the fundamental right, it becomes a luxury and that is like comparing the public transport vehicle to having a BMW; Everyone has to stop on the same road, everyone has to comply with the traffic rules. In other words, I think that both prepaid and POS are either contributory and subsidized regimes; They must have the same rights of efficiency, effectiveness and access, therefore the result must be the same. The issue of prepaid is a luxury in the system that is in a commercial issue and an issue of competition and marketing, but the right to health should not discriminate against capacity, because we see that those who obtain prepaid are for a matter of economic capacity, so they should not have any privileges" AD01 - "As long as it does not interfere with others, if an EPS has prepaid, it must have its infrastructure without taking over that of other people, that is: that the principle of equity in the standard is complied with. If I have a pathology that requires a technology and I require it now, I should have it now and not because I am paying more because that is the need I have. Now it can be postponed because it is not prioritized or urgent and you can do it in two months, so be it, and if you want to bring it forward you go to your prepaid medicine but without using the resources that are intended for the common people, but that it is really something different." AD04 - "It seems to me that it is a fragmentation of service, or delay in care, as a barrier that many times the EPS in order to lengthen the resources that come from the health system for the compensation of its users, what they do is postpone appointments or put them in a long time and in that process many diseases advance that are not treated in time. So it seems to me that it is something reprehensible, very good for those who have prepaid, but very reprehensible for those who are in the subsidized regime, it should not happen. It should have the same management for all regimes and it should not be allowed by the ministry or by the national superintendence of health, to put a stop to it and control this situation that I know many EPS do as long as they do not overcongest and waste resources so quickly." AD05 - "That it shouldn't be like this because ultimately we all have the right to health. But since it is optional for each person to sign up or not, that has an optional cost that must also be taken into account when providing the service." TD01 - ". Now, I suppose doctors leave in their agendas appointments for policies only or prepaid and that definitely facilitates access. That shouldn't happen, the agenda should be open every day for everyone and you could get the appointments no matter what benefit plan covers it, but that's not the truth. And that's what you take when you buy insurance, agility... But it shouldn't be." TD02 - "To me, that seems to be a matter of inequity because health is a fundamental right and so we are bringing health to a market and supply condition... then the one who has more pays more and has more benefits; and sometimes not even the one who has the most because many people have prepaid and make an economic effort because they know that with that they can access certain services and attention that the traditional system does not allow, so why if you pay more, better opportunities are provided, better conditions of access and accompaniment that the regular system does not allow, it would be necessary to work on the education of people, but there it is demonstrated that the system can be efficient "but for those who pay more"" TD05 - "That's like declaring yourself disabled, I think it's valid. I think that, queeee, that there are things that one pays for, to travel first class, to enter through the door, through a preferential door because in the end they are resources, that is not free, that is, that prepaid medicine or the one who pays in cash for the health system how they manage the resources, that is, it is money that is constant and resounding and I think that I agree that they have those benefits because finally the institutions must be adapting, they are adapting umm certain facilities and certain care for that type of patients, different, that is, when I say adapt is that there are spaces for that attention of them no, that that does not imply displacement although in the / in general terms what is being done is displacement, thus displacing the population from the general social security system, the normal one, whether the POS is subsidized or contributory" AD03 - "That's what I mean when I want to have things above everyone else. When that happens, it should cost me a little more, this is like the VIP lines everywhere, you're in that line not because you asked for it, but because you're paying something to be there. I think that in health it would be the same, I shouldn't have faster or priority access to a technology compared to another person if I'm paying the same. The important thing is that the resources do exist and that we are not neglecting the people we really should be caring for because we are giving them access to prepaid medicine. In other words, there must always be a differentiation between the resources of prepaid medicine and those of the common people. AD04 - "But the El Rosario Clinic in the Treasury, they structured it and defined it for prepaid medicine, so I say: You're not interfering in anything, that's what it was done for and there people pay more for that, that's why they take it there. Even that clinic is sometimes used to take people who do not have prepaid medicine but who need some service because the prepaid medicine does not give them enough to cover all the installed capacity, they would do the opposite: that they begin to close beds at the El Rosario clinic in the center to take people who are with prepaid medicine. That's like the interpretation I do." AD04 | - "In other words, as if the fact that you have the economic capacity and the possibility of accessing the system in a more timely manner, I think, that the opportunity for access should be without discrimination." AD02 - "And when you're at the POS they're telling you that the agenda fills up very quickly, but when it's prepaid, the agenda isn't full or the gap opens... Eh, because there's also a way to pay differently the professionals who, what, who serve, right? So in the agendas, the professionals who do medicine with prepaid and who also combine with the POS know how, how to open the holes, give them space because the percentage of profit is different" AD02 - The question I would ask myself is if that person has the money to pay for a prepaid medicine, right? So why don't you bring more to the system and be guaranteed opportunity?" AD02 - "No, I think it's appropriate because I don't criticize the prepaid system, and whoever has the conditions to pay for it, what I point out is that we cannot build an inequitable system in it and by the same market model, generate barriers to access to the most unprotected class, because health is a universal right. We are not talking about recreation, leisure, aesthetic condition, we are talking about a condition inherent to life, to the human being: the condition of health. Whoever has the economic capacity, well, let them have a good walk, a good house, a good car, that is part of the current economic model, but in the face of health conditions, I think that it must be crossed by other economic variables, by another analysis of sustainability, by other social and business criteria, to guarantee universality –That is a principle "The universality of the health service"–" TD05 - "From an individual level, for the case of this woman it is good, because she was fortunate enough to access it quickly and this is probably going to impact her health positively, from a social point of view, it is a bad indicator because that implies that health is a right that is conditioned to the ability she has to pay for it and that situation should be something dealt with within the ministry. If at some point the system manages to have a maturity in its structure, access and efficiency that manages to guarantee people access to fundamental services to take care of their health status, if that were to be achieved, I imagine that these services of complementary plans and private insurance should be oriented to community issues. as they already have it, but if it sounds a little worrisome from a Public Health perspective, that in order for you to access a technology for a Cancer with an adequate therapy, the ideal is that the cancer catches you with a private policy, that seems worrying to me on that side." TD04 - "It's a disaster that example because that shouldn't happen. So what do we do when we have patients who are asked for certain studies or technologies? We parameterize them so that they are studies that are delivered in x certain time; That is to say: –An ultrasound for a pelvic mass, it cannot be more than 15 days, you can tell me: –"I have a friend from that EPS and they did it after two months"– We have to look at those particular cases, but we try to make sure that the majority of the population has or does not have a policy or prepaid, or supplementary plan that behaves like a prepaid plan but is a portfolio of the same EPS; it's something adverse like saying to the patient: You're in the EPS and the service isn't so good, but take the pack and I'm going to give you some technologies with direct access so sometimes that can be Machiavellian, so in the face of that there can be a delay. I'm not going to talk to you about a prepaid because prepaid is a completely different business, but in front of it" TD03 - "That speaks of an imbalance at the social level, of inequality, but since we are in a capitalist society, that happens. I believe that they have more access to economic resources and greater purchasing power, and in the same way, they pay better and make greater contributions to the EPS. I see this with very good eyes, that those who are better able to pay can give a little more to the system with the principle of solidarity and that can help the others who are in the subsidized regime, but it is something beneficial to the system, they have better attention and better priority. I don't see it as something weird, I think it's something very positive." AD05 - "I consider that if I am paying as a user for a service, then I have to have quality in that service because I was generated by expectations. If you're telling me – because you have prepaid medicine – you pay me 300,000 COP a month in addition to what you pay in the health system and if you have a headache you go directly to the neurologist. But they are benefits that come at an additional cost to the user. So here the view cannot be from the health system because this is already a separate business as such." TD03 |

Relevant phrases in the Citizens group

| Context | Causes | Consequences | Actions and interactions |
| --- | --- | --- | --- |

| - So it's like... Yes, it's a privilege, very good for him to have it! TRUE? But really, if you think about it in the humanitarian field, it's absolutely unfortunate. For this very reason, because the State should, as ehhh, subsidize us with that. (PCTR-CD08). - Q2: I consider it appropriate that there be prepaid medicine, I consider it pertinent that there is prepaid medicine, however, that it is fair to have more timely care than others; I don't think it's fair, it's a paradox of the system that asked in this way if I think it's good, and I don't think it's good that some citizens have better access to medicine than others. (PCTR-CD12) - From my condition, that is to say, for me the prepaid health care is fine, but I consider that there is a great inequity and difference between those who do not have the means to pay or access that prepaid health and have to accommodate themselves to a health service that is not so comfortable or with the same benefits, so I would not know what to answer there because for those who have the means to pay for it, Well, great, but for those who don't have the means to pay for a prepaid policy, it's very bad because the State should guarantee compliance with health services for any citizen regardless of whether they have the resources to pay for a prepaid one or not. (PCTR-CD12) - To the extent that they can access better technologies, it is good for them, but in the IPSs the issue of whether it is a subsidized or prepaid EPS should not be prioritized because that is part of an economic dynamic that in the IPS it needs a certain cash flow and how the prepaid one pays it almost in advance. (PCTRPC10) - But if someone wants to pay, let them pay! But there are some ethical minimums that should be guaranteed in the system, because it is very paradoxical that they do not attend to you and there is no agenda for this specialist, but that same specialist does have a private agenda. Well, that is an aberrant thing, they are ethical dimensions, which have perverted the health system more and more specialists. PCTRCD17 - But if people have the money to pay for it [...] – even though they shouldn't. If you pay an EPS month by month, I think you should be given the fast service; without waiting there... "Come back in so many days and we'll give you the test" No! if one is paying, or the government in the subsidized one, it should be the same: Give good and timely attention. PCTRCD16 - That it shouldn't be like that because if you require a service it must be on time; Silver or no silver, it must be on time. The one at the top is the one that enjoys the best benefits and the other has to wait. PCTRCD16 - 2: In general, what I was saying, if the resources are for health, I don't know if they should invest in something else, but directly for what they are. As in other countries that have health systems with very good care where everyone is treated equally, they have privileged care. Let's just say that everyone there has the attention that we have here for paying a little bit more. (PCTR-CD07), - I think it's discriminatory, because that should be the same for everyone, both for those who pay for prepaid medicine and can have that facility, and for those who don't. It seems to me that it is exclusive, if you have money to pay for a prepaid one, you will supposedly receive better care or certain privileges, but I think that technology should be for everyone and should not exclude those who are in a prepaid system or not. PCTR-CD13 - That strikes me as a very sad inequality in terms of social class to a certain extent. PCTRCD17 - It is worth mentioning that individual freedoms always exist; So I can't stop buying a car because the other one can't, but that's an accessory issue; a vehicle. In the case of a right, it is necessary to resolve what is essential for everyone and those businesses that arise later, such as prepaid ones. PCTR CD17 - . In other words, I am not against prepaid medicine, but looking at it from the other side for those who are not there, it is unfair that it is prioritized for an economic issue rather than for the urgency that the patient has to make his diagnosis and thus his timely treatment. PCTRPC10 - PK: No, that [laughs]... that shows that health is a business in this country and in the world... Well, I think that's what it is, right? Paying for prepaid medicine is just that, that health is a privilege and that it is definitely also a business... ehhhh (PCTR-CD08) | - PK: Ummmmm... that saved the patient's life [nervous laughter]... ehhhhh, well, the prepaid medicine shortened/shortened her path and suffering... and possibly the negative evolution of his disease... Or I don't, because I don't know if it's said like that, but well, that his illness could have worsened, but it's also to the detriment of those who have to wait the two months because they don't have the money to pay for the ultrasound. (PCTR-CD08) - I think that prepaid medicine came about because you want to have that quality of life that you are better cared for, that you are in better situations. PCTRCD14 - Because of the delays in the health system, when you are already of a certain age, you have access to prepaid medicine or for those loved ones that you do not want to suffer. PCTRCD14 | - From my condition, that is to say, for me the prepaid health care is fine, but I consider that there is a great inequity and difference between those who do not have the means to pay or access that prepaid health and have to accommodate themselves to a health service that is not so comfortable or with the same benefits, so I would not know what to answer there because for those who have the means to pay for it, Well, great, but for those who don't have the means to pay for a prepaid policy, it's very bad because the State should guarantee compliance with health services for any citizen regardless of whether they have the resources to pay for a prepaid one or not. (PCTR-CD12)      - But if someone wants to pay, let them pay! But there are some ethical minimums that should be guaranteed in the system, because it is very paradoxical that they do not attend to you and there is no agenda for this specialist, but that same specialist does have a private agenda. Well, that is an aberrant thing, they are ethical dimensions, which have perverted the health system more and more specialists. PCTRCD17 - But if someone wants to pay, let them pay! But there are some ethical minimums that should be guaranteed in the system, because it is very paradoxical that they do not attend to you and there is no agenda for this specialist, but that same specialist does have a private agenda. Well, that is an aberrant thing, they are ethical dimensions, which have perverted the health system more and more specialists. PCTRCD17 - In other words, you have a prepaid one and it's as if a king arrives, like: "Hey, the one who pays the prepaid arrived, so let's take care of him quickly because he pays a little more" because it seems to me that it shouldn't be like that because we should all have access to health, let's say privileged. Not that if you pay more you have more benefits, suddenly in some things it will apply, If you are going to eat very well, you have to invest money. But let's say that health should be at the service of citizens, and that everyone can access it without paying an extra cap to be treated with more privileges. I think it's a little bit wrong. (PCTR-CD07) - So look, that's very sad, it's like the one who wants the best care, they just pay more and they don't have the most affordable installments that you say like: look how rich, I'm going to pay for this prepaid! There are people who don't have 400,000 pesos of what you normally pay; Somebody's going to say, "That's what I'll do in a month!" - , it is a very sad panorama because people die, that is, there are people who do not want to live and enjoy life very little, but there are some who do value it too much, they are young and want to live or have projects, and one of those things happens that simply because of having a bad health system that person dies and that is it and perhaps they had plans that they could not carry out; And just because you had money you were saved, I find that very sad. (PCTR-CD07). |
| --- | --- | --- |

I think that prepaid medicine is that alternative when the State in the provision of services "And I am talking about the State from the EPSs to the IPSs" in the care in quality and time, does not cover (CD-09)

1. Juan Felipe Araújo, father of the twins: "I have been told that there is no scientific evidence to prove that the drug will be effective. However, there is also no evidence to the contrary. A father would seek the treatment for his daughters." (El Espectador, July 7, 2018) [↑](#footnote-ref-1)
